# Comparison of two inspiratory muscle training protocols in people with spinal cord injury: a secondary analysis

**DOI:** 10.1038/s41394-023-00594-2

**Published:** 2023-08-12

**Authors:** Anne E. Palermo, Jane E. Butler, Claire L. Boswell-Ruys

**Affiliations:** 1 Neuroscience Research Australia, Sydney, NSW Australia; 2 University of New South Wales, Sydney, NSW Australia; 3 Prince of Wales Hospital, Sydney, NSW Australia

**Keywords:** Translational research, Health services

## Abstract

**Study design/setting:**

Secondary analysis.

**Objectives:**

To compare the change in maximal inspiratory pressure (PI_max_) over the first 4 weeks of two different inspiratory muscle training (IMT) protocols and explore if either method is more effective for people with spinal cord injury.

**Methods:**

Data originated from two published studies. Participants completed flow-resistive IMT (F-IMT) at 80% daily PI_max,_ 7 days/week (supervised weekly), or threshold IMT (T-IMT) at 30–80% weekly PI_max,_ twice-daily, 5 days/week (supervised every session). Seven participants from each trial were matched by training adherence, level of spinal cord injury, impairment grade (A–C), and height. Differences between F-IMT and T-IMT groups in training intensity, breaths taken, inspiratory work, and the change in the PI_max_ from baseline at the end of week four were analysed.

**Results:**

Over 4 weeks, there was no difference in the change in PI_max_ between groups (Absolute change in PI_max_ (cmH_2_O): *p* = 0.456, Percent change in PI_max_ relative to baseline: *p* = 0.128). F-IMT participants trained at a higher intensity (median: 77 vs 22 cmH_2_O, *p* = 0.001 and 80% baseline vs 61% baseline, *p* = 0.038) but took fewer breaths (840 vs 1404 breaths, *p* = 0.017) than T-IMT participants. Inspiratory work was similar between groups (64,789 vs 65,910 (% PI_max_ × number of breaths), *p* = 0.535).

**Conclusions:**

Our findings support both methods of IMT as the change in PI_max_ and inspiratory work were similar between groups. However, daily high-intensity F-IMT with intermittent supervision, required fewer breaths and less participant and therapist time. Future studies should examine optimal dosage and supervision required to achieve increased PI_max_.

## Introduction

Respiratory complications are a leading cause of morbidity and mortality for people with a spinal cord injury (SCI) [1]. Respiratory muscle weakness, which is a common sequela of the biomechanical and neurological changes associated with SCI [2], is predicted to be the primary cause of these respiratory complications [2]. One option to combat respiratory muscle weakness is respiratory muscle training (RMT). RMT encompasses interventions targeted to improve respiratory muscle strength and function including: inspiratory muscle training (IMT) [3, 4], expiratory muscle training (EMT) [5], combined IMT and EMT [6, 7], singing [8], breathing with abdominal weights [9], abdominal functional electrical stimulation [10], and iso- or normo-capnic hyperpnoea [11]. Two meta-analyses report significant positive effects of RMT for people with SCI [12, 13]. IMT protocols may be of greatest benefit because maximal inspiratory pressure (PI_max_) best predicts the risk of developing pneumonia during the initial rehabilitation stay[2] and improvements in PI_max_ are associated with a decreased risk of experiencing a respiratory complication [7]. Despite the evidence, the clinical uptake of IMT protocols is still lacking and respiratory compromise remains a leading cause of disability and death in this population [1].

Multiple factors may limit the clinical translation of IMT protocols found in the literature. One such debated factor is the dose, or the work of breathing, completed by the trainee during the program [12, 13]. Traditionally, work of breathing is calculated by multiplying the pressure produced by the volume of air moved. For the purposes of IMT, where each breath is to total lung capacity, work is influenced by (1) training intensity, defined as either the raw pressure or the percent of baseline PI_max_ reached during training, and (2) training volume, estimated by the number of breaths completed during the IMT program. Total training volume can be derived from the number of training days per week, the number of training sessions per day, the number of breaths per session and the volume of the breaths. Training frequencies of three to seven days per week are regularly reported, with recent trials reporting three to five training days weekly, but breaths per session and sessions per day vary [6, 7, 12–14]. The optimal IMT program has yet to be identified.

Another factor that limits clinical translation of IMT protocols is the varied types of IMT devices. Threshold resistance training (T-IMT) is the most widely reported mode of IMT used by people with SCI [12, 13]. Threshold devices contain a spring-loaded valve that opens to allow airflow when the trainee reaches the set pressure threshold during an inspiration. Flow-resistance training (F-IMT) devices have also been used to train people with SCI [3, 4]. F-IMT devices have small, fixed openings that create resistance as the trainee inspires. Faster inspiratory flow rates create greater pressure resistance in the F-IMT devices [15]. These two training modes have not been directly compared in the general population of people with SCI [16], leaving clinicians to guess which type of device is the most appropriate for their patients.

The clinical translation issues related to work and mode of IMT are compounded by the amount of supervision provided during trials and the lack of adherence reporting. Two studies investigating RMT in athletes with SCI did not provide fully supervised sessions and reported high adherence but these findings may not be generalizable to the non-athlete population [6, 17]. Most RMT studies provide fully supervised sessions which is not feasible in a community setting. Further, many trials report the target volume and intensity of IMT initially prescribed to participants but fail to report the actual adherence to those training variables. Neither of the recent meta-analyses of RMT in people with SCI report the adherence to the proposed protocols of included studies [12, 13]. To compare training programs or prescribe IMT clinically, clinicians and researchers need a better understanding of the inspiratory work that was achieved by participants in trials.

The current difficulties in between-trial comparison and the absence of program adherence data could limit RMT translation to clinical practice. A potential solution to these issues is to instead compare the actual inspiratory work that study participants complete; that is, the intensity of training based on either the absolute training pressure in cmH_2_O or the percent of baseline PI_max_ at which breaths are performed multiplied by the total number of breaths completed. This exploratory secondary analysis compared two different IMT protocols based on the inspiratory work performed throughout training. The primary goal of these exploratory analyses was to identify a superior IMT program based on the change in PI_max_. Second, we compared training variables to identify factors that influence the efficacy of SCI-based IMT paradigms across two trials. Last, we investigated relationships between self-reported exertion and intensity; a significant relationship would aid clinicians when prescribing IMT without a PI_max_ measurement. All exploratory analyses aimed to improve the clinical translation of IMT for people with SCI.

## Methods

### Study characteristics

Inspiratory muscle strength outcomes in two published original IMT studies in people with chronic SCI were compared. One study [4] used F-IMT (PrO2, PrO2Fit, Smithfield, RI, USA) and the second [7] used T-IMT (Threshold-IMT, Philips Respironics, Chichester, UK). Both studies had approval from affiliated human research ethics boards and were registered prior to trial commencement (NCT04210063; ANZCTR12612000929808). Informed written consent was obtained from participants prior to initiating study activities in accordance with the Declaration of Helsinki.

Both studies assessed maximal inspiratory pressure (PI_max_) at baseline prior to the start of IMT. The PrO2Fit was used to assess PI_max_ in the F-IMT study from residual volume in a seated posture at baseline and each day that a participant trained. The Hyp’Air Pulmonary Function Test (Medisoft, Sorinnes, Belgium) was used to measure PI_max_ from functional residual capacity in a seated posture at baseline while the MicroRPM (CareFusion, UK) was used to measure PI_max_ weekly during the T-IMT study. In the F-IMT study 11 participants with chronic SCI completed F-IMT at 80% of a daily PI_max_ for a target frequency of 7 days/week in their homes, supervised once weekly for 4–20 weeks, with variability in duration due to changes in the study protocol during the COVID-19 pandemic [4]. In the T- IMT study, 30 participants with acute SCI, and 32 participants with chronic SCI completed 2 daily supervised T-IMT sessions in the hospital (participants with acute SCI) or in their homes (participants with chronic SCI, >1 year duration of injury) 5 days/week for 6 weeks [7]. The T-IMT group trained at 30–80% PI_max_ assessed at the start of each week. Inclusion and exclusion criteria and training protocols for each study are shown in Table 1. In both studies, participants were instructed to train with breaths to total lung capacity (TLC).

**Table 1:** Protocol descriptions.

	Flow inspiratory muscle training group (F-IMT) (*n* = 11) [4]	Threshold inspiratory muscle training group (T-IMT) (*n* = 62) [7]
Inclusion Criteria	• Aged ≥18 years • Spinal cord injury of any neurological level of injury • American Spinal Injury Association (ASIA) Impairment Scale (AIS) grades A, B or C defined by the International Standards for Neurological Classification of Spinal Cord Injury (ISNCSCI) • ≥1-year post-initial injury date	• Aged ≥18 years • Spinal cord injury-induced tetraplegia between C4 and C8 with related respiratory deficits • AIS grades A, B or C defined by the ISNCSCI • Medically stable as deemed by treating physician • ≥4 weeks (acute, *n* = 30) or ≥1 year (chronic, *n* = 32) post-initial injury date
Exclusion Criteria	• Mechanically ventilated • Pregnancy • Current use of Beta-blockers or using pacemakers • Acute respiratory complication, pressure sore, or urinary tract infection • Individuals who could stand	• Mechanically ventilated • Pregnancy • Significant chest trauma such as flail ribs or pneumothorax • Diagnosis of a major co-existing respiratory or neurological illness or a cognitive impairment
Training protocol	• Training intensity set at 80% daily PI_max_/SMIP/ID • 7 sets of 6 breaths, once daily • 7 days per week • 4–20 weeks of training • Once weekly supervision	• Initial training intensity was 30% PI_max,_ adjusted weekly by a physiotherapist- up to 80% of a weekly PI_max_ • 3–5 sets of 12 breaths, twice daily • 5 days per week • 6 weeks of training • All training sessions supervised
Training Targets in first 4 weeks	28 sessions	40 sessions
	1176 breaths	2400 breaths

*PI*_max_ maximal inspiratory pressure, *SMIP* sustained maximal inspiratory pressure, *ID* inspiratory duration.

This table highlights the eligibility criteria, protocols, and target number of training sessions and breaths during the first four weeks of each protocol.

Seven of the 11 participants in the F-IMT study had chronic tetraplegia and were matched with active IMT participants with chronic SCI in the T-IMT trial by neurologic level of injury (and motor level or zone of partial preservation), AIS grade, height, and training adherence (% target sessions completed; Table 2). Complete data were available for all 14 participants for 4 weeks. Therefore, comparisons of the training protocols were made for the first 4 weeks of training.

**Table 2:** Matching characteristics.

	Flow inspiratory muscle training group (F-IMT) [4]				Threshold inspiratory muscle training group (T-IMT) [7]			
Matched pairs	NLI	AIS	Height (cm)	Adherence	NLI	AIS	Height (cm)	Adherence
1	C7	C	182	50%	C3	C	180	65%
2	C5	A	185	100%	C4	B	182	100%
3	C4	B	154	89%	C4	B	180	80%
4	C4	A	170	54%	C4	B	178	65%
5	C3*	B	182	96%	C5	B	180	88%
6	C6	A	175	43%	C7	A	186	65%
7	C1*	A	175	93%	C6	B	180	90%

Pairs of participants were matched on Neurologic Level of Injury (NLI) and American Spinal Injury Association Impairment Scale (AIS) or motor level and zone of partial preservation if an appropriate match could not be found. (*) represents individuals matched based on motor level instead of NLI. The motor levels of the F-IMT participants in the 5th and 7th matched pairs were C5 and C7, respectively. Individuals were also matched based on height and adherence to their respective training protocols, represented as a percent of sessions completed.

### Outcome measures

The following variables were calculated for each study group over 4 weeks of training: median Intensity of IMT (Intensity-absolute (PI in cmH_2_O) and Intensity-% (%PI_max,_ PI normalized to baseline PI_max_)); the number of training breaths completed (#Breaths); and the calculated inspiratory work done (Work-absolute = Intensity-absolute × #Breaths and Work-% = Intensity-% × #Breaths). Adherence was calculated from the number of sessions completed/number of sessions prescribed in each protocol. Perceived effort, or rate of perceived exertion (RPE), during training was measured using the modified Borg Scale in the T-IMT trial and a 0 to 10 visual analog scale (0- not difficult to 10- the most difficult) in the F-IMT trial, and the medians were reported. Modified Borg Scale scores have been reported to have a linear relationship to the VAS (slope of 0.98) when used to measure dyspnea during arm crank activity [18], thus, a direct comparison of the RPE scores from the two IMT protocols was used.

At the end of 4 weeks of training, we measured post-IMT inspiratory muscle strength and calculated the change in absolute PI_max_ (ΔPI_max_, PI_max_ after 4 weeks training minus PI_max_ at baseline), change in PI_max_ normalized to baseline PI_max_ (%ΔPI_max_). We calculated the predicted change in absolute PI_max_. The predicted change in absolute PI_max_ was calculated based on the model from Raab et al.: Expected PI_max_ change (cmH_2_O) = [7 × (absolute PI_max_ at baseline/100)] × [(Median Intensity-%)/10] [19]. We also calculated the proportion of the expected ΔPI_max_ represented by the actual ΔPI_max_.

### Data and statistical analysis

Medians (IQ ranges) were calculated for baseline characteristics such as age, weight, duration of injury, and baseline PI_max_, as well as training related variables from the data of each participant in both groups. Mann–Whitney *U* tests were used to analyze between group differences for all baseline measures and all outcomes (mannwhitneyu from scipy.stats in Python v. 3.9.7). Exploratory Spearman Correlations were used to analyze relationships among work-absolute, work-%, ΔPI_max_, and %ΔPI_max_ across groups to further recognize factors that may impact protocol efficacy (spearmanr from scipy.stats in Python v. 3.9.7). Additional exploratory analyses included investigating the relationship between RPE and either Intensity-absolute or Intensity-% of the IMT. Significance was set at *p* < 0.05. No a priori power analysis was completed as this was an exploratory secondary analysis of a subset of previously published data from two studies. We did not perform Bonferroni corrections due to the exploratory nature of the study and the small sample.

## Results

Descriptive characteristics of the 14 participants selected for the matched data comparison are shown in Table 3. Characteristics used as criteria for matching did not differ significantly between groups (height or adherence), however people in the F-IMT group were younger (*p* = 0.002), had a shorter duration of injury (*p* = 0.002), weighed less (*p* = 0.030), and had a higher PI_max_ (*p* = 0.041) at baseline than the T-IMT group (Table 3). Further descriptive information about the overall trial samples can be found in the original publications [4, 7].

**Table 3:** Descriptive baseline characteristics of the two groups of participants matched for height and adherence.

Baseline characteristics	Total sample	F-IMT	T-IMT	*p* value
N	14	7	7	na
Females (N)	1	1	0	na
NLI (range)	C1–C7	C1–C7	C3–C6	na
AIS (range)	A–C	A–C	A–C	na
Height (cm)	180 (175–182)	175 (172–182)	180 (180–181)	0.437
Adherence (%)	84 (65–92)	89 (52–95)	80 (65–89)	0.949
Age (years)	43 (35–49)	34 (30–38)	49 (48–52)	0.002*
Duration of Injury (years)	21 (8–31)	6 (4–15)	31 (29–34)	0.002*
Weight (kg)	69 (58–74)	57 (54–69)	75 (68–83)	0.030*
Baseline PI_max_ (cmH_2_O)	77 (32–108)	97 (85–112)	31 (21–53)	0.041*

(*) denotes significant difference between groups in Mann–Whitney *U* analysis (*p* < 0.05).

Median (IQ range) values reported. Bolded variable names were used to match participant data between the two studies.

*NLI* neurologic level of injury, *AIS* American Spinal Injury Association (ASIA) Impairment Scale, *F-IMT* flow- inspiratory muscle training, *T-IMT* threshold- inspiratory muscle training, *PI*_*max*_ maximal inspiratory pressure.

**Fig. 1:**
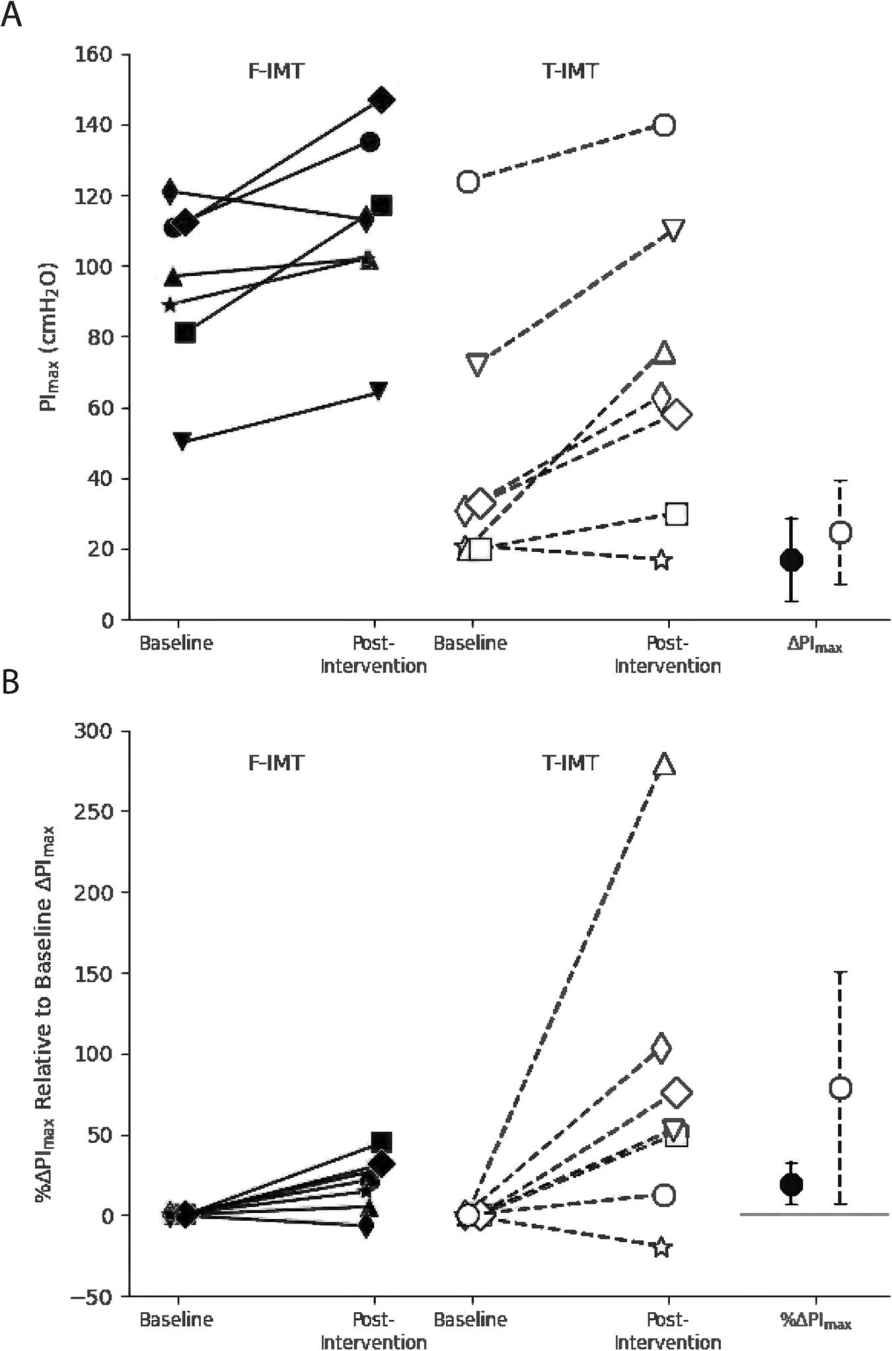
Change in maximal inspiratory pressure. **A** Individual absolute PI_max_ (cmH_2_O) at baseline and after 4 weeks of IMT and (**B**) individual %ΔPI_max_ (relative to baseline PI_max_) from the Flow-Inspiratory Muscle Training (F-IMT, solid lines, and solid markers) and Threshold-Inspiratory Muscle Training (T-IMT, dashed lines and open markers) groups (*n* = 7 in each group). Matched participants are represented by the same marker shape. The mean group changes and 95% confidence intervals are shown on the right of each panel for ΔPI_max_ in (**A**) and %ΔPI_max_ in (**B**). The 95% Confidence Intervals (95%CI) overlap between groups but do not overlap 0 cmH_2_O or 0%.

After 4 weeks of IMT the PI_max_ increased from baseline for both groups by a mean of 17.0 (95% CI: 5.2–28.9) and 24.7 (10.1–39.3) for the F-IMT and T-IMT groups, respectively (right panel Fig. 1A). However, no between-group difference was found in the absolute ΔPI_max_ or %ΔPI_max_ relative to baseline (*p* = 0.456 and *p* = 0.128, Table 4 and Fig. 1B). The PI_max_ of the two groups was not significantly different after training (*p* = 0.073), although it was half the size in the T-IMT group. Interestingly, the actual ΔPI_max_ as a proportion of the expected ΔPI_max_ was lower in the F-IMT group compared to the T-IMT group (*p* = 0.026, Table 4).

**Table 4:** Training protocol characteristics and training outcomes for the two groups of matched participants measured as an average over 4 weeks and after four weeks of IMT training.

Training characteristics and outcomes	F-IMT	T-IMT	*p* value
Total number of Breaths Completed over 4 weeks of IMT (#Breaths)	840 (609–1071)	1404 (1194–1710)	0.017*
IMT Intensity-absolute over 4 weeks of IMT (cmH_2_O)	77 (69–86)	22 (16–28)	0.001*
IMT Intensity-% over 4 weeks of IMT (% baseline PI_max_)	80 (74–88)	61 (47–70)	0.038*
Work-absolute over 4 weeks of IMT (breaths × Intensity-actual in cmH_2_O)	52479 (49971–69563)	38299 (23541–47322)	0.004*
Work-% over 4 weeks of IMT (breaths × Intensity-%)	64789 (48896–82122)	65910 (54932–128982)	0.535
RPE over 4 weeks of IMT (out of 10)	7.0 (6.0–8.2)	3.5 (2.7–3.9)	0.001*
Post-Intervention PI_max_ (cmH_2_O)	113 (102–126)	63 (44–93)	0.073
Absolute ΔPI_max_ (cmH_2_O, change from baseline PI_max_)	14 (9–29)	25 (13–35)	0.456
%ΔPI_max_ (ΔPI_max,_ relative to baseline PI_max_) (%)	22 (10–30)	53 (31–90)	0.128
Expected ΔPI_max_ (cmH_2_O, calculated from Raab et al. [19])	53 (49–61)	15 (10–20)	0.001*
Actual compared to expected ΔPI_max_ (%)	38 (19–49)	150 (94–182)	0.026*

(*) denotes significant difference between groups in Mann–Whitney *U* analysis (*p* < 0.05).

Median (IQ range) values reported. All medians and IQ ranges were derived from individual level data. *PI*_*max*_ maximal inspiratory pressure, *RPE* rate of perceived exertion, Expected PI_max_ change (cmH_2_O) = [7 × (absolute PI_max_ at baseline/100)] × [(Median Intensity-%)/10] [19], *F-IMT* flow- inspiratory muscle training, *T-IMT* threshold- inspiratory muscle training, *PI*_*max*_ maximal inspiratory pressure.

**Fig. 2:**
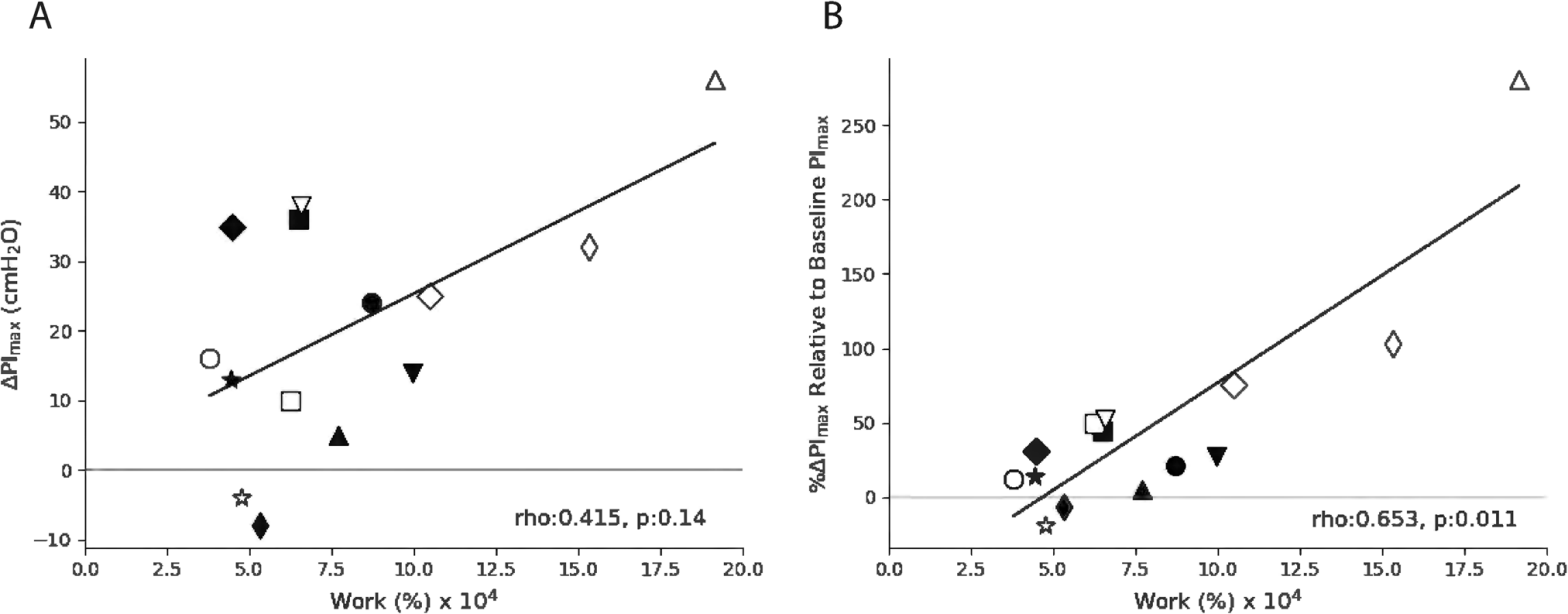
Correlations between Work-% and changes in PI_max_. Correlations between Work-% (calculated by multiplying the number of training breaths completed by the intensity of training as a percent of baseline maximal inspiratory pressure (PI_max_)) and (**A**) absolute change in PI_max_ (ΔPI_max_, cmH_2_O) and (**B**) %ΔPI_max_, (%ΔPI_max_ relative to baseline PI_max_). Participants in the Flow-Inspiratory Muscle Training (F-IMT) group (*n* = 7) are represented by solid markers while participants in the Threshold-Inspiratory Muscle Training (T-IMT) group (*n* = 7) are represented open markers. Matched pairs are plotted in the same marker shape. *p* < 0.05 indicates significant correlation.

**Fig. 3:**
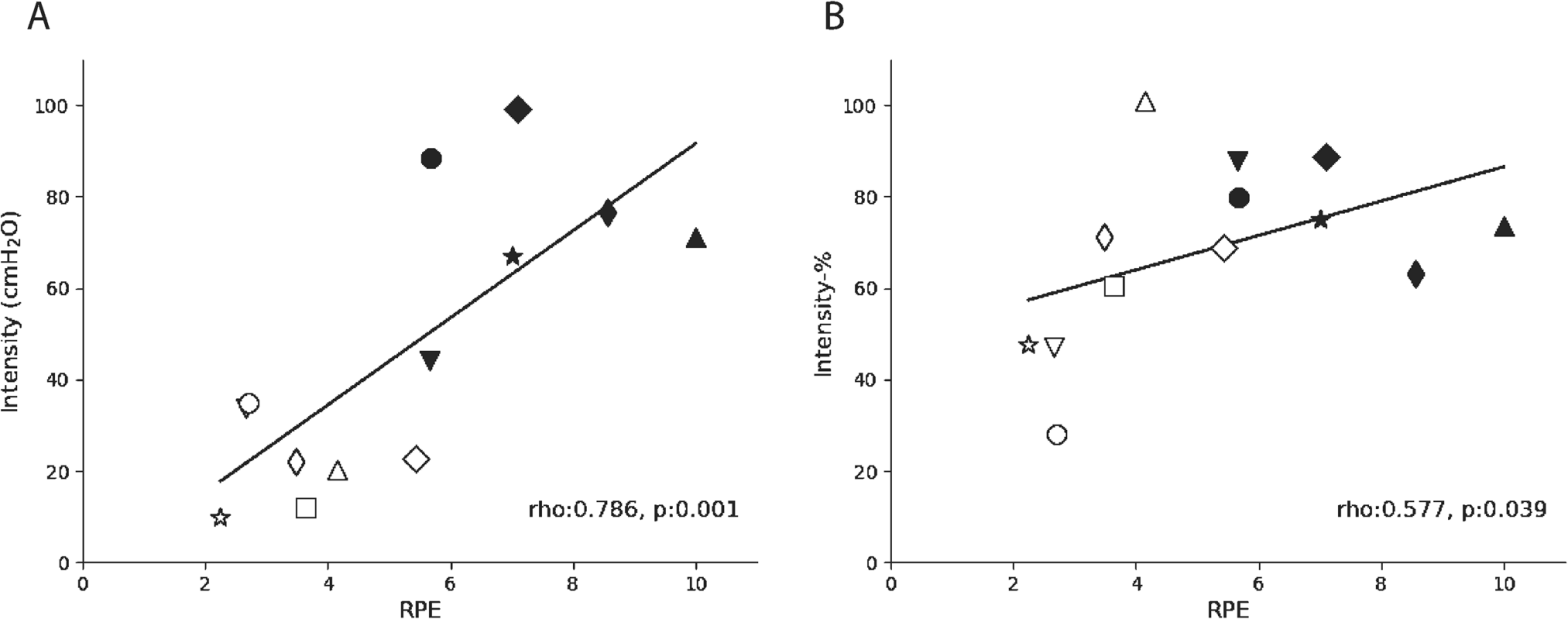
Correlations between perceived exertion and training intensities. Correlations between rate of perceived exertion (RPE) and (**A**) Intensity-Absolute and (**B**) Intensity-% (calculated as a percent of baseline maximal inspiratory pressure). Participants in the Flow-Inspiratory Muscle Training (F-IMT) group (*n* = 6, one person was not compliant with RPE reporting) are represented by solid markers while participants in the Threshold-Inspiratory Muscle Training (T-IMT) group (*n* = 7) are represented by open markers. Matched pairs are plotted in the same marker shape. *p* < 0.05 indicates significant correlation.

There was no significant difference between groups for Work-% performed over the 4-weeks, although Work-absolute was higher in the F-IMT group due to the higher baseline PI_max_ used to calculate Intensity-absolute (Tables 3 and 4). Spearman correlations found a significant positive relationship between Work-% and % ΔPI_max_ (rho = 0.665, *p* = 0.013) but not absolute ΔPI_max_ (rho = 0.429, *p* = 0.114) (Fig. 2, panels B and A, respectively). RPE had a significant positive relationship to both training Intensity-absolute (rho = 0.786, *p* = 0.001) and Intensity-% (rho = 0.577, *p* = 0.039) (Fig. 3, panels A and B, respectively).

## Discussion

This is the first analysis to compare the efficacy of two IMT protocols that used different devices and training programs in the general population of people with chronic tetraplegia. While both training protocols improved PI_max_ after 4 weeks of training, the findings of this secondary analysis do not identify a superior IMT protocol based on ΔPI_max_ or %ΔPI_max_. However, the findings do identify factors that may impact overall IMT protocol efficiency and show relationships that are clinically important. We found a positive correlation between Work-% performed and %ΔPI_max_, regardless of the device or protocol used. The Work-based findings are evidence that the Work-% construct may be used when comparing IMT protocols. Further, the relationship between perceived exertion and intensity of training may help clinicians grade IMT training intensity in the absence of facilities to assess inspiratory pressure or PI_max_.

After 4 weeks training, no group difference was found in %ΔPI_max_ or ΔPI_max_, despite the large difference in PI_max_ between the F-IMT and T-IMT groups at baseline, where the PI_max_ of the F-IMT group was more than three times that of the T-IMT group. However, the post-intervention PI_max_, ΔPI_max_ and %ΔPI_max,_ although not different between groups, in the F-IMT group were 1.8, 0.6, and 0.4 times the median values for the T-IMT group, respectively. The lack of significant differences may be due to the low numbers of participants included in the comparison analysis, as well as large inter-participant variability in PI_max_ resulting in an underpowered analysis and possible Type 2 error. Similar to our current findings in SCI, IMT training in people with chronic obstructive pulmonary disease (COPD) increased PI_max_, but with no difference in ΔPI_max_ after F-IMT compared to T-IMT [15]. Additionally, between group differences in ΔPI_max_ were not found in a study investigating T-IMT vs F-IMT vs no IMT in elite rugby athletes, most of whom had SCI [16]. However, it is unclear whether a significant increase in PI_max_ occurred within groups based on the analyses reported and the small numbers in each group (T-IMT group, *n* = 4; F-IMT group, *n* = 5; no IMT control group, *n* = 7)[16]. Therefore, while IMT in either form can increase PI_max_, further research is needed to identify if there is an optimal mode of training or training protocol for each diagnostic population.

The similar improvements in ΔPI_max_ or %ΔPI_max_ across F-IMT and T-IMT in the current study suggest that supervision of every training session may not be mandatory in every case to obtain positive results. All IMT sessions were supervised in the T-IMT group, while only one session per week was supervised in the F-IMT group. There are financial, transportation, and staffing barriers that may limit the clinical translation of fully supervised training protocols [20, 21]. However, supervision may be required for individuals with hand function impairments if adaptations are not available to allow for independent use of devices. Some form of clinical supervision is likely beneficial even for individuals with the ability to perform IMT independently or with assistance from a carer. Both the original F-IMT and T-IMT studies included follow-up phases where no supervision was provided. During the non-supervised phase, only 1 of 3 active participants (33%) and 16 of 62 participants (26%) continued to train in the F-IMT and T-IMT studies, respectively [4, 7]. No supervision seems to be detrimental to continued training after an exposure to supervised IMT in people with SCI. Similarly, a meta-analysis investigated the impact of supervision on adherence of people with non-neurologic chronic disease during follow-up exercise programs after participants had completed a 4–6 week supervised exercise program [22]. A pooled and weighted analysis of two studies found the proportion of people who were “partially adherent” to a home exercise program without any supervision was low (29%). This reported proportion is very similar to the unsupervised adherence rates reported by the studies included in this secondary analysis [4, 7, 22]. Future studies should continue to investigate supervision models, including remote supervision, that are sustainable and tailored to the individual abilities of people with SCI and clinicians, more importantly so in the era of telehealth consultations and treatments.

Beyond supervision, other training factors differed between the F-IMT and T-IMT groups including a higher number of breaths (#Breaths) taken in the T-IMT group with a lower training intensity. Overall, these differences did not result in significantly different ΔPI_max_ or %ΔPI_max_ outcomes in the current study. Raab et al. have reported on the predictive relationship of training Intensity-% and ΔPI_max_ in a retrospective study of an inpatient cohort (*n* = 67) with SCI ranging from C4-T12 levels of injury (AIS A-D) [19]. They found that median training Intensity-% and PI_max_ at baseline, but not #Breaths, were predictive of the ΔPI_max_ after a median of 6 weeks (interquartile range of 5–8 weeks) of training [19]. The expected ΔPI_max_ for each group in the current study (based on the Raab et al (2019) equation) indicated that the T-IMT group performed much better than expected (167%) while the F-IMT group performed much worse than expected (26%) [19]. These differences from expected changes in each group raise doubt about the utility of this predictive equation. The equation may be inaccurate when comparing community dwelling individuals with variable injury characteristics, training devices, protocols including number of sessions, intensities of training, duration of training and levels of supervision. Our limited data cannot determine the utility of the equation and variability of sample and training factors is not accounted for in previous meta-analyses [12, 13].

Across all 14 participants in the current study, there was a strong positive correlation between Work-% (calculated from #Breaths × Intensity-%) and %ΔPI_max_, regardless of the training paradigm. Work was not included in the model by Raab et al. [19] despite work being a predictor of increased strength in limb resistance training [23, 24]. However, work is not commonly reported in IMT trials [12, 13], and in the current study, we have calculated work relative to baseline PI_max_ across the first 4 weeks of training only. The relationship of Work-% and %ΔPI_max_ is unknown beyond this. Nevertheless, the strong correlation suggests that higher levels of work produce higher %ΔPI_max_ even if the Intensity-% of training is reduced and the #Breaths is increased to compensate. This occurred for the T-IMT group, in which the Intensity of training relative to baseline (Intensity-%) was 30% of that for the F-IMT group. The calculation of work relative to PI_max_ at baseline (Work-%) could allow comparison of the impact of different IMT protocols to a common outcome (%ΔPI_max_) and may offer a more complete analysis of the effect of IMT on respiratory function in people with SCI. Future studies could report Work-% to improve between-protocol comparison and translation to clinical practice as well as allow for tailored approaches to IMT.

The data from the current study also showed that across both groups, training intensity (both Intensity-% and Intensity-absolute) are positively correlated with RPE, a measure of the effort required to do the training. Similar relationships between effort and pressure have been reported previously in able-bodied people [25], people with COPD with and without anxiety [26], and people with chronic tetraplegia [27]. Further, a meta-analysis reported that peak oxygen uptake and peak power output improve when individuals with SCI complete perceptually regulated exercise protocols [28]. Although IMT protocols were not included in that meta-analysis, our findings support that perceptually regulated IMT may be effective at determining the dose for intensity of IMT via RPE scores since a respiratory pressure meter is not always available in clinical settings. Clinicians may be able to prescribe the Intensity-% of IMT based on RPE; for example, from our limited data in Fig. 3, training at an RPE of 5 is likely to represent a training intensity of at least 65% PI_max_. The relationship between Intensity% and RPE suggests that individuals with tetraplegia can generally perceive the intensity at which they are performing IMT and warrants further investigation to confirm.

The introduction of the Work-% variable, calculated from training Intensity-% and #Breaths, and the recognition of the correlation between perceived exertion and training intensity are clinically important and should be considered in future research. However, this secondary analysis is limited by its small sample size (and low power) and the existing baseline differences in study participant groups. In general, the participants in the F-IMT study were younger and had a shorter injury duration than participants in the T-IMT groups. Older age and longer injury duration are related to poorer respiratory function [29–31] which may have contributed to the lower average baseline PI_max_ of participants in the T-IMT study. The higher baseline PI_max_ of the F-IMT group may have resulted in a ceiling effect in ΔPI_max_.

## Conclusion

This novel secondary analysis compared outcomes from F-IMT and T-IMT protocols in individuals with chronic tetraplegia. Both the protocols elicited similar improvements in PI_max_ after 4 weeks of training and therefore, the most efficacious protocol could not be determined. This may be due to significant differences between groups in PI_max_ at baseline and a relatively small sample size. However, the findings suggest that in-person supervision may not be required for all IMT sessions. The positive correlation between perceived effort and training Intensity-absolute and Intensity-%, support the use of perceived effort as a potential surrogate for Intensity-% when clinicians prescribe IMT without the ability to monitor inspiratory pressure or PI_max_. This prescriptive relationship is especially important since Work-% is derived from Intensity-% and increased Work-% was related to increased PI_max_ after 4 weeks. Measurement of Work-% may be used in future research or clinical practice to help researchers and clinicians to compare IMT protocols and to determine the best options for people with SCI. We recommend that future IMT clinical trials should report adherence and Work-%, where possible, to improve generalizability and comparisons between protocols.

## Supplementary information

Reproducibility checklist

## Data Availability

Data use requests in line with the ethical approval of the initial studies submitted to the corresponding author will be considered for approval.
